# Bending Fatigue Properties of Ultra-High Toughness Cementitious Composite (UHTCC)

**DOI:** 10.3390/ma17133128

**Published:** 2024-06-26

**Authors:** Pengju Wang, Kaijian Huang, Gong Shen, Yixin Miao, Jiansheng Wu

**Affiliations:** 1 College of Civil Engineering, Nanjing Forestry University, Nanjing 210037, China; 18251851497@163.com; 2 Wuxi Communications Construction Engineering Group Co., Ltd., Wuxi 214000, China; gongshen0526@163.com (G.S.); yixinmiao269@163.com (Y.M.); 3 Nanjing Kegong Coal Science and Technology Research Co., Ltd., Nanjing 210037, China; wujiansheng@njsjy.com

**Keywords:** Ultra-High Toughness Cementitious Composite, toughness, bending fatigue characteristics, fatigue damage

## Abstract

Ultra-High Toughness Cementitious Composite (UHTCC) represents a composite material meticulously engineered on the foundation of micromechanical principles. The multi-crack cracking and strain-hardening characteristics of UHTCC enable it to be applied to orthotropic steel decks to control the crack width. Different from most studies which only focus on hybrid fiber or fatigue characteristics, this paper studies the influence of hybrid fiber content on static mechanical properties, flexural toughness, and flexural fatigue characteristics of UHTCC under different stress levels. The compressive and flexural strength, bending toughness, and fatigue damage of UHTCC under different fiber ratios were compared, and the fatigue properties of hybrid fiber UHTCC were verified. The results reveal that hybrid fiber exerts a more pronounced effect on toughness, augmenting the maximum folding ratio by 23.7%. Single-doped steel fiber UHTCC exhibits a characteristic strain-softening phenomenon attributable to inadequate fiber content, whereas the bending toughness index of hybrid fiber UHTCC surpasses that of SF1.5P0 by 18.6%. Under low-stress conditions, UHTCC demonstrates a nearly threefold increase in bending fatigue life with a mere 1% steel fiber content, while the influence of polyvinyl alcohol (PVA) fiber on fatigue life is more significant: with an increase of only 1/5 volume content, the fatigue life increased by 29.8%, reaching a maximum increase of 43.2% at 1/4 volume content. Furthermore, the fatigue damage accumulation curve of UHTCC follows a three-stage inverted S-shaped trajectory. The inclusion of PVA fiber facilitates early initiation of stable cracking during the fatigue failure process, thereby advancing the entire strain stability development stage and mitigating external load forces through the proliferation of micro-cracks. Consequently, compared to SF1P0, the ε_0_ of SF1P5 experiences a significant increase, reaching 143.43%.

## 1. Introduction

Compared with the flexible paving layer, the rigid paving layer, represented by cement-based materials, significantly improves stress conditions within the paving layer. Consequently, the adoption of rigid paving layers to address steel bridge deck paving issues has emerged as a research focal point [1,2,3,4]. Tong [5] conducted experiments and utilized finite element models to perform parametric analyses. Both experimental and simulated results indicate that UHTCC layers substantially enhance the toughness and durability of composite bridge floor structures. Wang [6] investigated various peg spacings and longitudinal reinforcement ratios to analyze the deformation and cracking characteristics of composite structures. It was observed that the cracking stresses σ_0.05_ and σ_0.1_ of UHTCC steel bridge panels were 58.6% to 216.8% and 58.9% to 213.5% higher, respectively, than those of ordinary steel concrete bridge panels.

The road and bridge structures are subjected to various loads, and when the stress reaches the limit, deformation occurs, leading to fatigue failure [7,8,9,10]. UHTCC exhibits a similar fatigue failure phenomenon as ordinary concrete, albeit with differing fatigue life and characteristics [11,12,13,14]. This disparity has spurred scholarly investigation into the fatigue performance of UHTCC [15,16,17], particularly focusing on bending fatigue characteristics, which has yielded fruitful results [18,19,20,21,22]. Ma [7] reinforced RC beams with steel plates and UHTCC, finding that the bending resistance of RC beams containing a UHTCC layer and steel plate was more than double that of the control group. After 200,000 and 2 million fatigue loads, the residual bearing capacity was 96.2% and 94.2% of the static load test, respectively. Regarding the optimization of the composite material itself, aside from altering the composition of the binding material [23,24], a method more suited to the design mechanism of UHTCC involves focusing on the fiber material [25,26,27]. This includes mixing fibers with varying stiffness, elastic modulus, or scale to enable different fibers to function optimally at different stress stages, thereby enhancing the mechanical properties of the composite materials [28,29]. Yang [30] developed a multi-scale reinforcement system using millimeter-level PE fiber, micro-scale calcium carbonate whisker, and nano-scale carbon nanotubes as externally doped fibers. The results indicated that calcium carbonate whisker exerted the greatest influence on compressive strength, while polyethylene fiber had the greatest impact on bending and tensile strength. When the contents of the three additives were 1.55%, 2.17%, and 0.154% respectively, the composite material exhibited optimal comprehensive performance.

As mentioned above, all the studies only focus on hybrid fibers or fatigue properties, and the exploration of hybrid fiber UHTCC is insufficient. This paper aims to improve the static mechanical properties of UHTCC, study its bending fatigue properties, explore the effect of hybrid fiber content on the static mechanical properties and high cycle fatigue characteristics of UHTCC, optimize the mix ratio design of composite materials, and play a certain guiding role for practical engineering.

## 2. Materials and Experiment

### 2.1. Experimental Materials

The materials used in this work are P·O 42.5 ordinary silicate cement (Conch, Anhui, China), silica ash (Langtian, Sichuan, China) with an apparent density of 1290 kg/m^3^ and a specific surface area of 21,500 m^2^/kg, standard sand (Iso, Xiamen, China), tap water, polycarboxylate superplasticizer (Nanjing, China), and stabilizer produced by Germany Mingling Chemical (Essen, Germany). Short straight copper-coated steel fiber and PVA fiber (Shandong, China)were vital in this study. PVA fiber and steel fiber have large differences in elastic modulus and physical size. The mechanical properties of the two fibers were obtained through a drawing test and physical property test, as shown in Table 1 and Table 2.

### 2.2. Methods

The research group has already conducted relevant research [31]. In addition, some more mature studies also provide guidance for the experimental design of this paper [32,33]. The specific mixing ratio adopted in this paper is shown in Table 3. The mass fraction of the cementation material is 10%; the volume content of steel fiber is0%, 1.0%, and 1.5%; the volume content of steel fiber of blended fiber UHTCC is 1.0%; and the volume content of PVA fiber is 1/5, 1/4 and 1/3 of the former, respectively. They are named SF0P0, SF1P0, SF1.5P0, SF1P5, SF1P4, and SF1P3.

**Figure 1:**
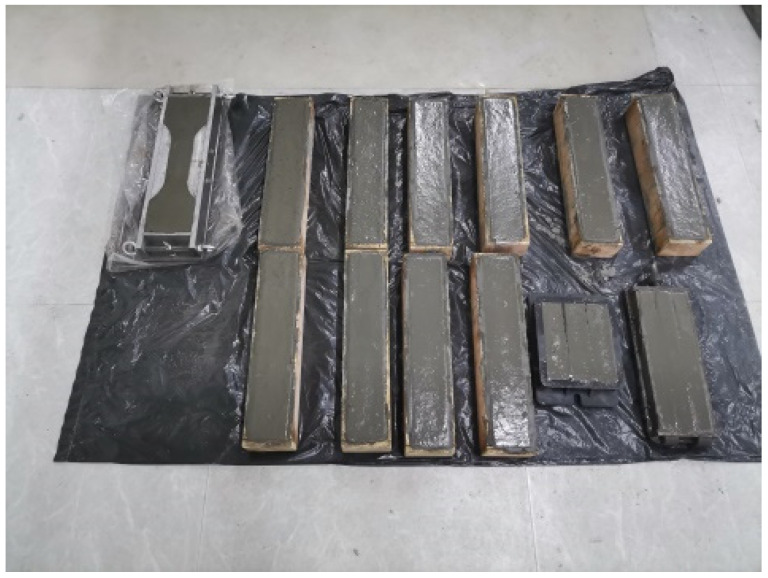
Specimen molding.

By referring to relevant specifications [34,35], specimens for flexural strength, compressive strength, and three-point bending fatigue tests were prepared. The sample sizes and number of each group are shown in Table 4. The specimen pouring molding is shown in Figure 1. The mold is released after 28 days of maintenance under standard conditions.

**Figure 2:**
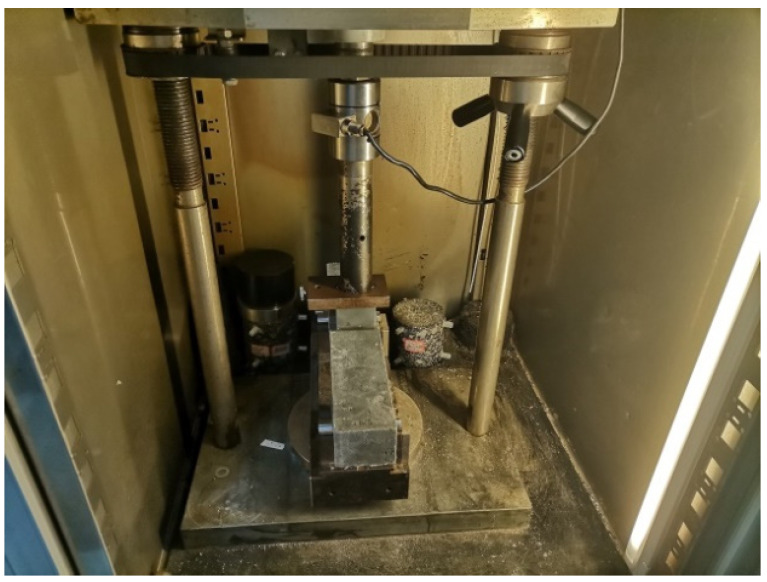
Pneumatic servo universal material testing machine.

In this paper, the data results were processed by Origin software (9.90.225), a compressive and bending strength testing machine was used for the strength test, and the static load test data of three-point bending were obtained from the CMT4204 electronic universal testing machine (Sansi Eternal Technology, Ningbo, China). Finally, the bending fatigue test was carried out by a pneumatic servo universal material testing machine (Sansi Eternal Technology, Ningbo, China), as shown in Figure 2.

**Figure 3:**
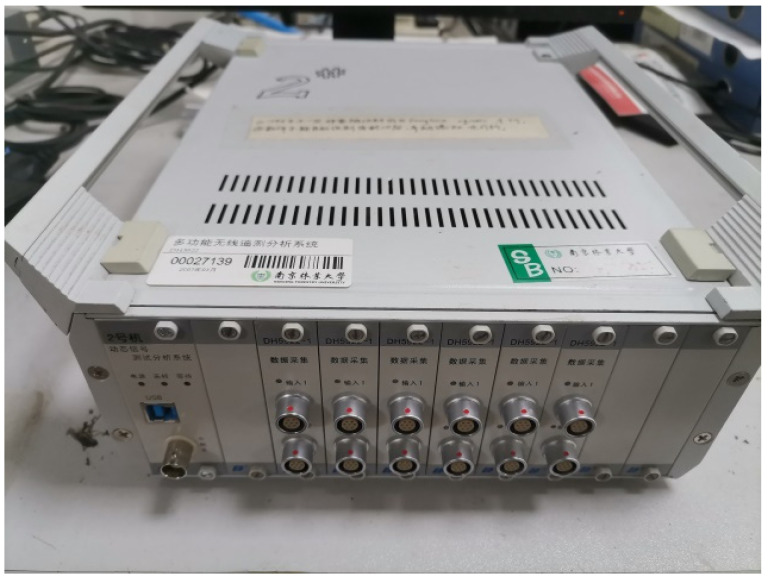
DH5922 strain test system.

In the fatigue test, the static load strength is the average value of the four test blocks in each mixing ratio, and the parameters selected for the fatigue life are the loading frequency of 5 Hz. Thus, the loading frequency does not affect the fatigue life measured by the fatigue test [31]. The loading waveform is selected as a sine wave. Another parameter of the fatigue test is the stress level, that is, the ratio of the maximum stress during the cycle to the static load strength, taking 0.6 and 0.7. The strain data in the fatigue test were obtained from the DH5922 strain testing system, as shown in Figure 3. Considering the large discreteness of the fatigue test, three parallel samples were prepared for each group of tests, and their average values were taken as the final fatigue life results.

## 3. Results and Discussion

### 3.1. Influence of Fiber Content on Static Mechanical Properties

**Figure 4:**
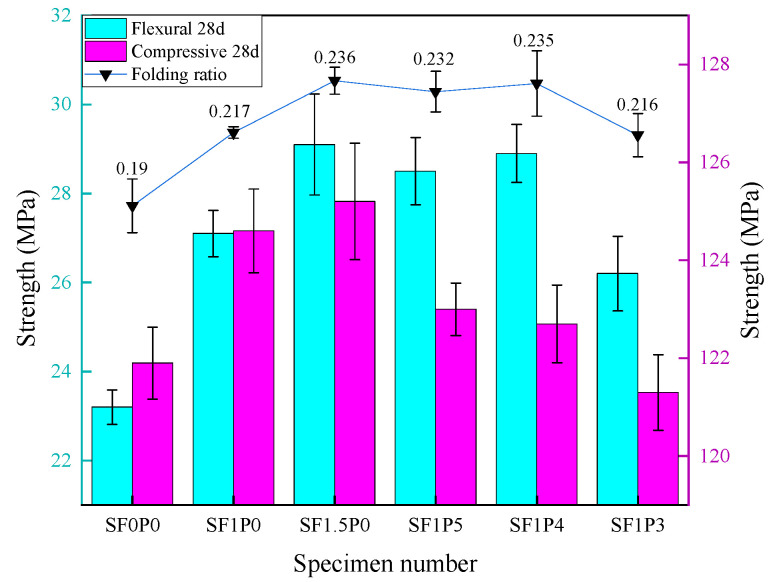
Effect of fiber content on mechanical properties of UHTCC.

UHTCC strength test results are shown in Figure 4. Compared with SF0P0, the compressive strength of SF1P0 increases by 2.2%, and it increases by only 0.5% when steel fiber is added to 1.5% volume content. It can be observed that the enhancement of compressive strength with single-doped steel fiber tends to saturate near 1.0% volume content while playing a pivotal role in improving bending strength. The flexural strength of SF1P0 and SF1.5P0 increases by 16.8% and 27.6%, respectively. The impact of steel fiber on UHTCC compressive strength manifests primarily in two aspects. Firstly, it reinforces the matrix and restrains the transverse deformation of UHTCC specimens. Secondly, it bridges micro-cracks that develop continuously alongside cracks during compression failure, effectively resisting external stresses.

**Figure 5:**
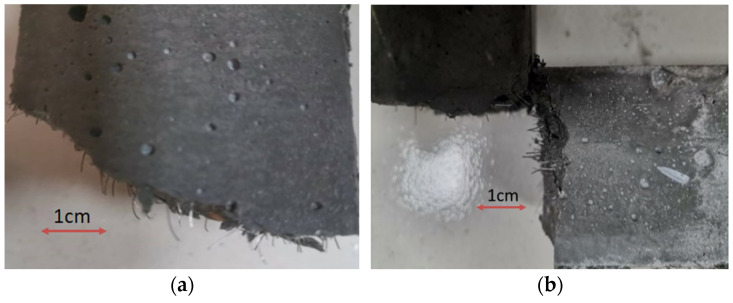
Section of steel fiber distribution. (**a**) Even distribution of steel fibers. (**b**) Uneven distribution of steel fibers.

Throughout this process, continuous adhesive slip occurs between the steel fiber and the matrix, thereby enhancing the compressive strength of the specimen to a certain extent. However, owing to the low water–binder ratio employed in this study, the compressive strength of the specimen itself is relatively high, resulting in an insignificant increase in compressive strength from the addition of steel fiber, especially when the distribution of high-volume doped steel fiber is excessively concentrated (as depicted in Figure 5). This concentration forms a low bonding strength interface between the fiber and the matrix, slowing the growth of compressive strength. Simultaneously, as the volume content increases, the number of steel fibers per unit volume increases while the average spacing between fibers decreases. This phenomenon leads to the increase in steel fibers at cracks in the UHTCC matrix, where they can better distribute and transfer stress, thereby delaying crack development and improving flexural strength. Moreover, during the process of pulling out steel fiber, overcoming the bonding force between the matrix and the fiber requires additional work and consumes more energy. Macroscopically, it is evident that within a certain range, a higher steel fiber content results in a more pronounced improvement in the folding effect.

The enhancement of UHTCC’s mechanical properties through the incorporation of PVA fiber is constrained. On the foundation of a 1.0% steel fiber content, the introduction of PVA fiber leads to a maximum 6.6% increase in bending strength, a growth rate lower than that observed with SF1.5P0. Additionally, the inclusion of PVA fiber induces a slight reduction in compressive strength, primarily attributed to its flexible nature and low elastic modulus. The mechanism of action involves densification of the structure through self-filling. Excessive PVA fiber content or inadequate dispersion leads to aggregation within the cement matrix, forming agglomerates that create weaker interfacial transition zones, thus diminishing compressive strength. During UHTCC specimen formation, fibers establish a stable framework within the cement matrix, effectively mitigating external loads. Upon structural damage, such as fracture or pull-out of steel fibers, substantial energy is dissipated, thereby suppressing crack propagation. Notably, the hydrophilic properties of PVA fiber significantly mitigate cracks induced by water evaporation and hydration, thereby enhancing concrete bending strength. However, the analysis of experiment results presented herein suggests the superior efficacy of singly doped steel fiber.

The flexographic ratio, representing the ratio of flexographic strength to compressive strength, serves as an indicator of material toughness. Analysis of the data depicted in Figure 4 reveals a comparable increase in the folding ratio of the two fibers within the specimen. Relative to the undoped fiber, SF1P0 exhibits a 14.2% rise in the bending ratio. Upon introduction of PVA fiber, the bending ratio peaks at 0.235 for SF1P4, marking a 23.7% surge, akin to the impact observed with SF1.5P0. However, given the slightly lower quantity of PVA fiber compared to steel fiber, the hybrid fiber demonstrates enhanced toughening properties. Compared with Huang’s research [28], the compressive strength of the specimen prepared in this paper is insufficient, but under the condition of less fiber content, the bending strength of the specimen is more than 40% higher than the maximum value of 20.3 MPa of the former.

### 3.2. Effect of Fiber Content on Flexural Toughness

**Figure 6:**
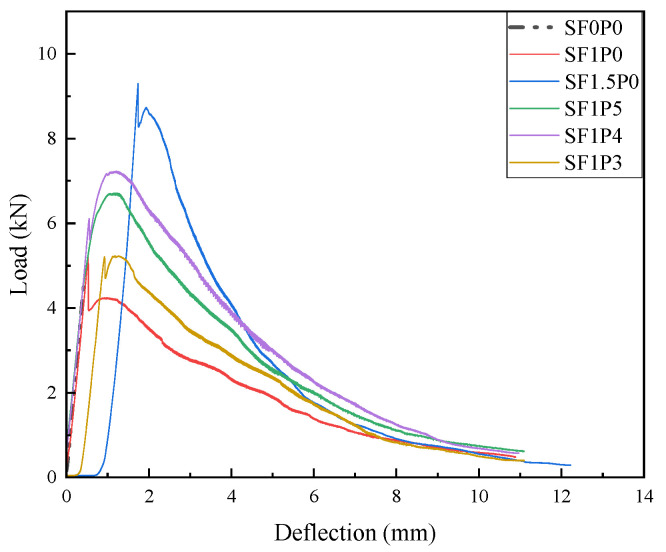
Deflection-load curve.

The load-deflection curves obtained from the three-point bending toughness test are plotted in Figure 6. The bending toughness index TIF is obtained according to the area enclosed by the curve and the X-axis to represent the bending toughness [36]. The specific data are shown in Table 5.

As depicted in Figure 6, the loading process of specimens reveals distinctive development trajectories in various UHTCCs. Notably, the curve representing SF0P0 exhibits a singular upward trend, indicative of brittle fracture. Conversely, the inclusion of steel fiber alters the behavior, as observed in the SF1P0 curve, which manifests three distinct stages. In the initial ascent, the specimen undergoes early loading, wherein the load sustained by the specimen escalates alongside deflection. Upon reaching a critical deflection threshold, the specimen experiences cracking. Transitioning into the yield phase, the presence of steel fiber mitigates rapid fracture, prolonging the specimen’s structural integrity. Cracks induced during this phase influence deflection, leading to fluctuations in load. In the subsequent descent phase, the deflection-induced separation between steel fibers and the matrix, coupled with crack propagation, contributes to load diminishment. Additionally, the analysis of the figure reveals that the maximum load in the rising section surpasses that in the yielding section. This discrepancy is attributed to inadequate steel fiber content, leading to a rapid decline in load between the rising and yielding sections. This decline aligns with the typical curve characteristics observed in strain softening of conventional steel fiber concrete [37]. The curve trajectories of SF1.5P0 and SF1P0 exhibit similar patterns, albeit with a more moderate decline observed between the rising and yield sections in the latter. The cracking deflection of the specimen experiences a significant surge from 0.53 mm to 1.74 mm, marking a 228% increase. Additionally, the bending toughness index escalates from 20.68 J to 28.45 J, denoting a 37.6% increment. These findings underscore that, despite the strain softening phenomenon, when compared to the SF0P0 specimen, the cracking deflection escalates by 314%, and the bending toughness index increases by more than 28-fold.

There is no abrupt transition between the ascending and yielding segments of the blended fiber curve, showcasing the characteristic strain-hardening properties of UHTCC. This observation, when coupled with the data in Table 5, reveals that the maximum cracking deflection of the mixed fiber specimen falls short compared to that of the singly doped steel fiber specimen, registering a decrease of 27.6% compared to SF1.5P0. This discrepancy primarily arises from the larger size and higher elastic modulus of steel fibers, which exert a more pronounced influence on macro-level crack suppression. Consequently, within a defined range, higher steel fiber content correlates with increased cracking deflection. Conversely, when a specific volume of PVA fiber is introduced, the bending toughness index of SF1P4 surpasses that of SF1.5P0 by 18.6%. This enhancement stems from the bridge and crack resistance properties inherent in PVA fibers, which impede the propagation of fine cracks surrounding the initial crack and restrict crack width expansion within a limited scope. Consequently, achieving failure through bending in the specimen necessitates greater force and entails increased energy consumption during the load failure process, thus resulting in a numerically higher bending toughness index for the hybrid fiber specimen.

Additionally, as the relative volume content of PVA fiber increases from 1/5 to 1/3, there is a mere 5% rise in the cracking deflection of the specimen, juxtaposed with a notable 25.1% decline in the bending toughness index. Furthermore, in the case of SF1P0, the augmentation of PVA fiber initially escalated the toughening effect before undergoing a subsequent decline, attributed to the poor dispersion of PVA fiber. Upon excessive addition of PVA fiber to the composite material, a mere 5% increase in the cracking deflection of the specimen is observed alongside a significant 25.1% decrease in the bending toughness index. This phenomenon is exacerbated by severe fiber clumping, posing challenges not only in micro-crack suppression post specimen formation but also in deteriorating the operational efficacy of the composite material. Such clumping also accounts for the diminished flowability of the specimen during the pouring process, further underscoring the adverse effects of excessive PVA fiber content.

### 3.3. Effect of Fiber Content on Bending Fatigue Properties

Factors such as the non-uniformity of concrete materials and the randomness of the fatigue process result in large discreteness of the measured data in the fatigue test. In order to obtain results that are not too conservative and more consistent with the actual project, this paper selects the density function of Weibull distribution to represent the distribution law of fatigue life N of UHTCC from the perspective of probability [38], and Weibull variable is represented by N_ξ_. Fatigue life N under the condition of a given survival rate P is calculated, as shown in Equation (1) [38].

(1) $$P\left(N_{\xi }>N\right)=\int_{N}^{\infty }f\left(N\right)dN=exp\left[-{\left(\frac{N-N_{0}}{N_{a}-N_{0}}\right)}^{b}\right]$$

In the equation, N_0_ is the minimum fatigue life, N_a_ is the characteristic fatigue life, and b is the Weibull shape parameter. For a simple operation, N_0_ = 0 is usually assumed to obtain the Weibull distribution of two parameters, and the natural logarithm is applied to both sides of the equation to obtain Equation (2) [38].

(2) $$\ln\left[\ln\left(\frac{1}{P}\right)\right]=b\ln N-b\ln N_{a}$$

The above two formulas are theoretical equations for the study of concrete fatigue. Traffic requires high cycle fatigue of pavement materials, so this paper uses a 0.6 stress level to study the high cycle fatigue characteristics of UHTCC at low-stress levels, and the average bending fatigue life of each group is shown in Table 6.

**Figure 7:**
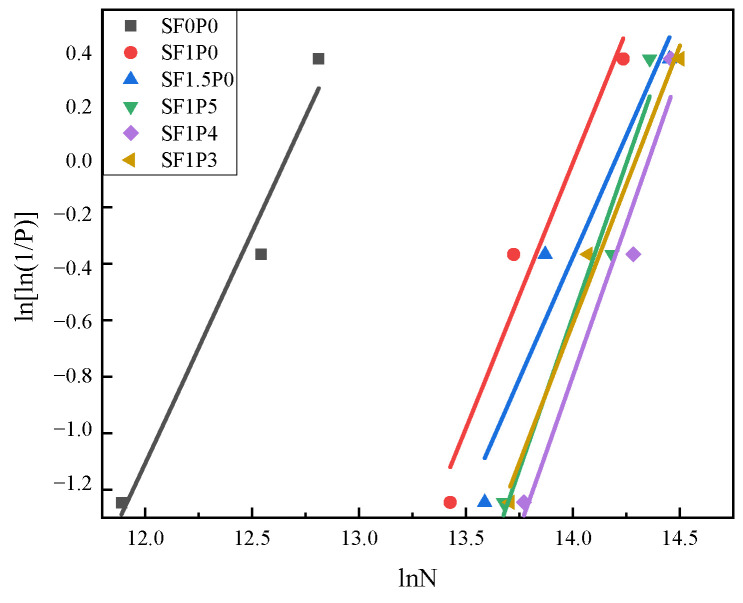
Weibull distribution test of fatigue life.

In this paper, there are three groups of UHTCC fatigue life tests for each ratio, and the assigned probability P according to the Weibull distribution is 0.75, 0.50, and 0.25. The linear fitting analysis of UHTCC fatigue life data is shown in Figure 7. The relevant parameters of the linear regression equation obtained by fitting are shown in Table 7.

As shown in Table 6, SF0P0 specimens experience a sudden brittle fracture, attributed to the short fatigue life of undoped steel fibers. Introducing 1% steel fibers extends fatigue life by nearly threefold, while for SF1.5P0, the increase is still notable at 20.4%. Notably, the addition of PVA fibers enhances fatigue life significantly, with a 29.8% increase at 1/5 volume addition, peaking at a 43.2% increase at 1/4 volume addition. Furthermore, as indicated in Table 7, the correlation coefficient R^2^ consistently hovers around 0.9, indicating a high degree of linear regression fit. The bending fatigue life of the composite material predominantly conforms to the Weibull distribution, as supported by the analysis of these parameters.

In the bending fatigue test, the maximum stress manifests within the mid-span of the specimen, where the initial crack propagates along the surface, culminating in fracture failure. This paper focuses on documenting the initiation time of cracks to assess the behavior of band cracks during composite fatigue failure. Given the minute nature of the initial cracks, both sides of the span are moistened with a wet cloth, and recording commences upon the detection of micro-cracks absorbing water. At stress levels of 0.6 and 0.7, recordings are taken, respectively, every 1 h and 0.5 h following the test initiation, and the fatigue life corresponding to crack formation time is computed. Additionally, this paper introduces the split working time ratio, as defined by Equation (3).

(3) $$R=\frac{N-T}{N}\times 100\%$$

**Figure 8:**
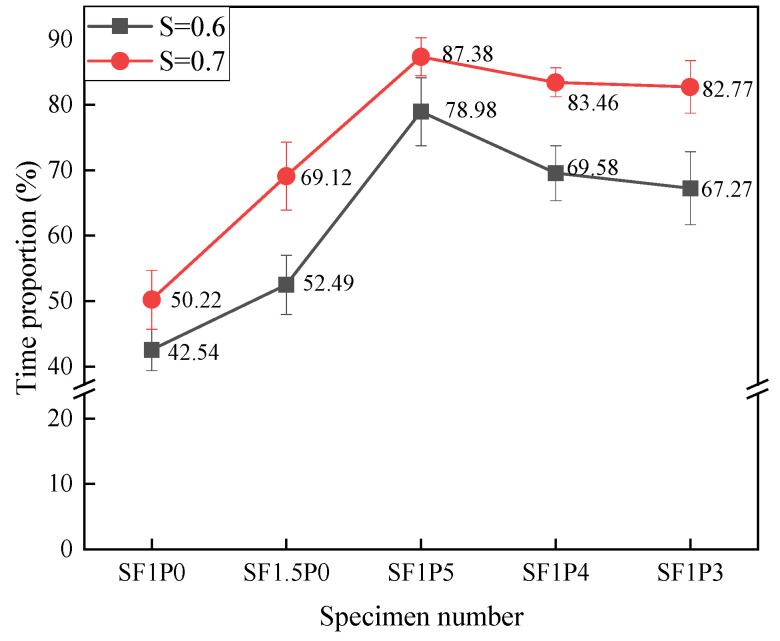
Proportion of UHTCC cracking working time under different stress levels.

In the equation, R is the proportion of working time with cracking, N is the fatigue life of the specimen, and T is the initial cracking time. According to the formula, the working time ratio of each group of specimens with band cracks was calculated as shown in Figure 8.

**Figure 9:**
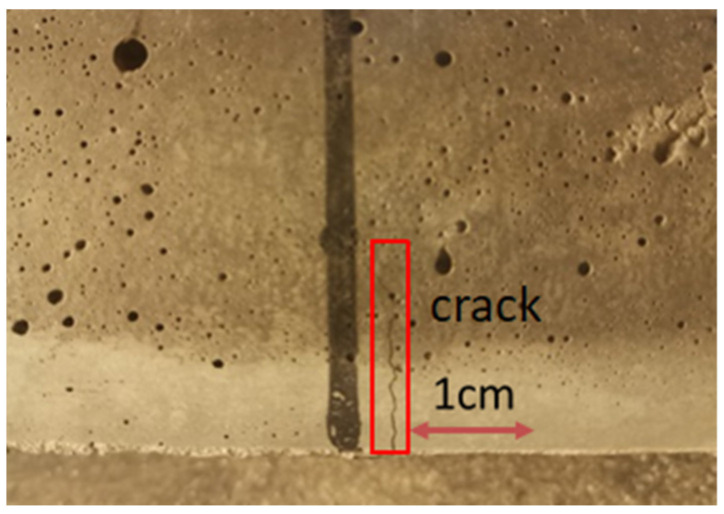
Cracks in single-doped steel fiber UHTCC under load.

**Figure 10:**
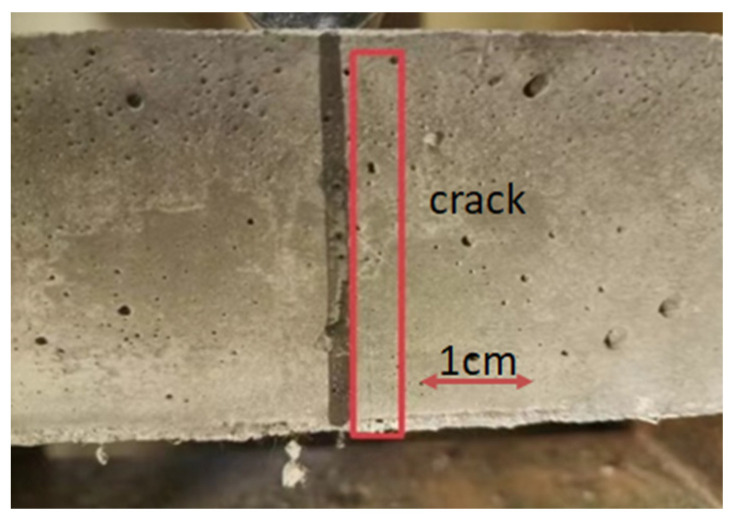
Cracks in mixed fiber UHTCC under load.

Based on the analysis depicted in Figure 8, under different stress levels, the time for the initial crack to appear decreases, and R also increases with the increase in stress level. This phenomenon arises due to the escalation of the maximum load across the span with increasing stress levels, resulting in earlier cracking of the specimen and bearing of greater stress through the formation of fine cracks. Moreover, the augmentation of stress level leads to varying increments in the *R* value across different specimens. Particularly noteworthy is the most pronounced average increase observed in SF1.5P0 at 16.63%, followed closely by SF1P3 at 15.5%. This trend can be attributed to the inclusion of fibers, notably PVA fibers, which not only bolstered toughness but also fortified the capacity to restrict micro-crack expansion, consequently expanding the crack space within composite materials. This augmentation facilitates earlier cracking of the material to withstand higher loads. Throughout testing, it can be observed that the cracking of UHTCC with single-doped steel fiber under load was distinctly visible, as illustrated in Figure 9, whereas the cracking observed in hybrid fiber configurations appeared more subtle, as depicted in Figure 10. This phenomenon has also occurred in other studies [39].

### 3.4. Bending Fatigue Damage Analysis

When subjected to bending fatigue loads, internal defects within concrete gradually propagate, accumulating damage until eventual failure. The progression of concrete fatigue damage unfolds in two discernible stages, contingent upon the emergence of macroscopic cracks. Initially, in the absence of macroscopic cracks, damage accrues through the expansion of micro-cracks within the cement matrix, as well as through fiber tension and frictional interactions with the matrix. Upon reaching a critical threshold, macroscopic damage manifests in concrete, resulting in the appearance of macroscopic cracks. Subsequently, in the second stage, post macro-crack formation, the damage primarily ensues from the extension and enlargement of the micro-crack zone within the concrete, alongside the progression of macroscopic cracks.

**Figure 11:**
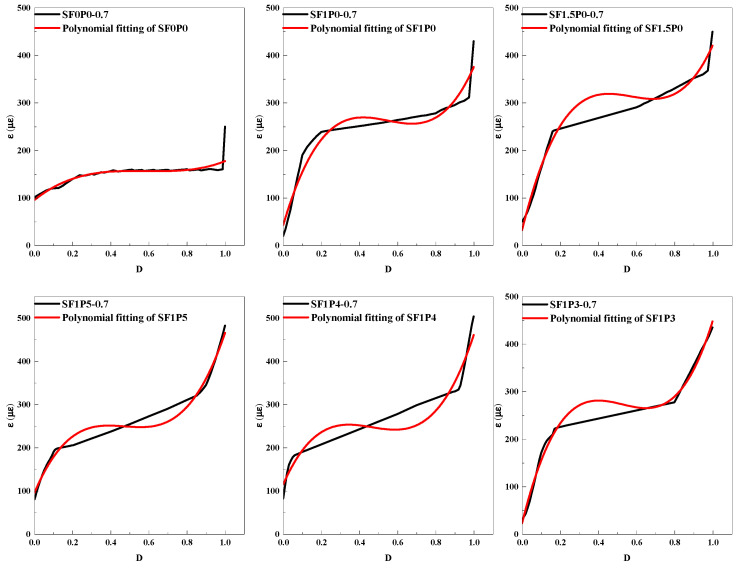
D-ε curve of UHTCC.

Given the remarkable attributes of UHTCC and their extensive fatigue testing period, this paper delves into the accumulation process of fatigue damage during the initial stage when the stress level is 0.7. Commencing from the onset of loading until the emergence of macroscopic cracks, the strain gauge positioned on the underside of the specimen fractures. This observation extends and elaborates upon the previously recorded time for the inception of micro-cracks, facilitating an evaluation of the material’s operational efficacy concerning crack development. Employing the symbol ‘D’ to signify the extent of damage and structural degradation, a defined range [0, 1] characterizes its magnitude. A value of 0 indicates the absence of fatigue-induced damage, while 1 denotes its presence. Leveraging the data acquired on the bottom strain of the specimen throughout the experimentation, a D-ε curve is constructed to scrutinize the correlation between UHTCC damage accumulation and strain, drawing on the Miner linear cumulative damage theory [40], as depicted in Figure 11. The maximum strain values portrayed in the figure represent the peak strains recorded at the specimen’s bottom during each cycle, as measured by the strain gauge.

The D-ε curve is nonlinearly fitted by a cubic polynomial to describe the damage and strain relationship of the whole fatigue process of UHTCC. When D = 0, there is no damage to the concrete, and the initial strain *ε_0_* exists. Failure occurs when D = 1, and the ultimate strain ε_f_ exists. Table 8 below shows the fatigue damage fitting equation and related parameters.

As can be seen from Table 8, R^2^ represents the correlation coefficient of the fitted curve. The correlation of the fitted equations in the table is all greater than 0.9, indicating that the curve-fitting effect is relatively good. As depicted in Figure 11, the overall trajectory of the curve exhibits an inverted S-shaped developmental pattern, delineated into three discernible stages. The initial phase represents a period of rapid development wherein cyclic loading initiates, prompting the emergence of initial defects within the concrete and subsequent rapid strain accumulation. Following this, the progression enters a phase characterized by stable development, wherein the damage increment sustains steady growth at a relatively constant rate. Subsequently, the trajectory transitions into an intense development stage, marked by a sharp escalation in fatigue strain concurrent with the evolution of damage until internal damage accumulation reaches its critical threshold, thereby placing the concrete in an unstable condition, culminating in failure. In comparison with SF0P0, the incorporation of fiber into UHTCC induces notable alterations. Firstly, during the initial stage, the ε_0_ value exhibits a diminutive magnitude but experiences accelerated development. This phenomenon stems from the fact that damage accumulation during this phase primarily arises from the expansion of initial defects within the concrete, a process effectively mitigated by the presence of fibers. Secondly, the introduction of fibers imparts a measure of toughness to UHTCC, thereby fostering a sustained upward trend in strain during the subsequent stage. Additionally, owing to the toughening influence of fibers, the ε_f_ value of UHTCC experiences a significant augmentation, registering an increase of up to 188.4%.

Quantitatively, the ratio of cycles corresponding to the conclusion of the first stage and the completion of the second stage to the total number of cycles was designated as k_1_ and k_2_, respectively. The comparative analysis reveals that the hybrid fiber UHTCC exhibits a relatively large k value, indicating a balanced progression across various UHTCC strain stability development stages. However, the inclusion of PVA fiber enables earlier engagement in structural reinforcement during the fatigue failure process of specimens, advancing the overall strain stability development phase. Compared with the data in the table, it is found that the initial strain increases significantly after adding PVA fiber. The inherent characteristics of PVA fiber dictate an earlier transition of specimens into the multi-crack failure mode to counteract external loading [18,20]. Consequently, the ε_0_ of SF1P0 and SF1P5 experiences a significant increase of 143.43%, while SF1P4 records an increase of 191.89%, albeit at a decelerated pace. Conversely, the ε_0_ of SF1P3 registers a notable decline, dropping even lower than SF1P0. This phenomenon arises from the incorporation of excessive PVA fiber, leading to a proliferation of initial defects. Although this results in greater inhibition of micro-crack expansion during the initial stage, it concurrently compromises various material properties, culminating in the emergence of dense cracks at lower strains. Nevertheless, the inclusion of PVA fiber significantly elevates the ε_f_ of the specimens, with SF1P5 showing a 16.9% increase compared to SF1P0.

## 4. Conclusions

In this paper, flexural and compressive strength tests, three-point bending static tests, and fatigue tests are carried out on fiber UHTCC with different contents to explore the influence of hybrid fiber content on the static mechanical properties and high-cycle fatigue characteristics of UHTCC, and the following conclusions are reached.

1. The hybrid fiber specimens obtained the same toughness as the single-doped steel fiber specimens with less fiber content. PVA fiber exerts a profound influence on both enhancing toughness and augmenting crack deflection in composite materials. The flexural toughness index of hybrid fiber UHTCC surpasses that of SF1.5P0 by up to 18.6%.
2. At a low-stress level, the fatigue life of PVA fiber increases more significantly, which reaches the highest increase of 43.2% at SF1P4. The working time ratio of UHTCC band cracks increases with the increase in stress level. The effect of hybrid fibers can be attributed to the suppression of various cracks in the matrix at various stages and the enhancement of the cracking space of the composite materials.
3. Incorporating mixed PVA fiber triggers early activation, facilitating stable cracking during the fatigue failure progression of specimens. This early activation advances the strain stability development phase and mitigates external load through the formation of additional micro-cracks. Consequently, the initial strain ε_0_ of SF1P5 is 143.43% higher than that of SF1P0. Compared with single-doped steel fiber, the ultimate strain of hybrid fiber specimen increases by 16.9%.

## Figures and Tables

**Table 1:** Basic properties of steel fiber.

Length (mm)	Equivalent Diameter (mm)	Density (kg/m^3^)	Fusing Point (°C)	Tensile Strength (MPa)
13.12	0.20	7800	1200	2868

**Table 2:** Performance parameters of polyvinyl alcohol.

Length (mm)	Equivalent Diameter (um)	Density (g/cm^3^)	Initial Modulus (MPa)	Elongation at Break (%)	Breaking Strength (MPa)
12	15	0.9	35.7 × 10^3^	19.47	1589

**Table 3:** Benchmark mix ratio.

Cementing Material	Water–Binder Ratio	Sand Binder Ratio	Water Reducer (%)	Stabilizer (%)
1	0.22	1.0	0.7	0.4

**Table 4:** Specimen size.

Test	Specimen Size	Number of Specimens
Flexural and compressive tests	160 mm × 40 mm × 40 mm	3
Three-point bending test	380 mm × 65 mm × 50 mm	4
Bending fatigue test	380 mm × 65 mm × 50 mm	6

**Table 5:** Bending toughness data of UHTCC.

Specimen Number	SF0P0	SF1P0	SF1.5P0	SF1P5	SF1P4	SF1P3
Cracking Deflection (mm)	0.42	0.53	1.74	1.20	1.21	1.26
Flexural toughness index (J)	0.98	20.68	28.45	30.37	33.74	22.74

**Table 6:** Bending fatigue life of UHTCC.

Specimen Number	SF0P0	SF1P0	SF1.5P0	SF1P5	SF1P4	SF1P3
Mean fatigue life (times)	264,132	1,037,359	1,248,526	1,346,539	1,485,994	1,391,845

**Table 7:** Linear regression equation-related parameters.

Specimen Number	SF0P0	SF1P0	SF1.5P0	SF1P5	SF1P4	SF1P3
$\mathrm{Regression}\;\mathrm{coefficient}\;\hat{a}$	−20.7894	−26.3432	−24.5201	−31.0026	−31.0606	−28.2101
$\mathrm{Regression}\;\mathrm{coefficient}\;\hat{b}$	1.6400	1.8785	1.7244	2.1724	2.1615	1.9712
R^2^	0.9450	0.9041	0.8625	0.9164	0.9139	0.9739

**Table 8:** D-ε fatigue damage equation.

Specimen Number	Fatigue Damage Equation	R^2^	ε_0_ (με)	ε_f_ (με)
SF0P0-0.7	ε = 262.28D^3^ − 379.32D^2^ + 178.99D + 99.52	0.9869	99.52	161.47
SF1P0-0.7	ε = 1430.70D^3^ − 2853.68D^2^ + 1781.96D + 39.35	0.9059	39.35	398.33
SF1.5P0-0.7	ε = 1618.98D^3^ − 2956.68D^2^ + 1726.26D + 32.02	0.9054	32.02	420.58
SF1P5-0.7	ε = 1038.12D^3^ − 2265.67D^2^ + 1597.43D + 95.79	0.9616	95.79	465.67
SF1P4-0.7	ε = 996.28D^3^ − 2296.80D^2^ + 1646.49D + 114.86	0.9505	114.86	460.83
SF1P3-0.7	ε = 1620.52D^3^ − 3262.98D^2^ + 2067.65D + 22.25	0.9713	22.25	447.44

## Data Availability

The original contributions presented in the study are included in the article, further inquiries can be directed to the corresponding author.
